# A Novel Sparse Array Configuration for Direction of Arrival Estimation with Increased Uniform Degrees of Freedom and Reduced Mutual Coupling

**DOI:** 10.3390/s24030808

**Published:** 2024-01-26

**Authors:** Shuang Wei, Gencun Zhu, Ying Su

**Affiliations:** College of Information, Mechanical, and Electrical Engineering, Shanghai Normal University, Shanghai 200234, China; weishuang@shnu.edu.cn (S.W.); 1000511938@smail.shnu.edu.cn (G.Z.)

**Keywords:** sparse arrays, uniform degrees of freedom, mutual coupling, difference coarray, direction of arrival estimation

## Abstract

Sparse arrays are widely employed in array signal processing due to their obvious advantages in array element distribution and uniform degrees of freedom (uDOFs). In this paper, a generalized augmented multi-subarray nested array (GAMSNA-I) and its variant, GAMSNA-II are proposed, with the objective of increasing uDOFs and reducing mutual coupling. Based on two subarrays of the prototype nested array (NA), GAMSNA-I is constructed by reconfiguring the dense uniform linear array (ULA) and forward-shifting the sparse ULA. GAMSNA-II is obtained by sparsifying the dense part of GAMSNA-I, ensuring constant uDOFs while further reducing mutual coupling. Subsequently, the closed-form expression for the uDOFs of GAMSNA-I with an arbitrary number of sensors is derived, and the proof is provided that the uDOFs of GAMSNA-II remain unchanged relative to that of GAMSNA-I. Compared to some existing array configurations, both GAMSNA-I and GAMSNA-II exhibit improved uDOFs, with GAMSNA-II achieving lower mutual coupling. Simulation results show the superior performance of the proposed GAMSNA-I and GAMSNA-II.

## 1. Introduction

Direction of arrival (DOA) estimation is a critical signal-processing technique extensively applied in fields such as radar, sonar, and wireless communication [1,2,3,4,5] to determine the relative direction of signal sources. Uniform linear arrays (ULAs) [6,7,8,9] are frequently used sensor array types for DOA estimation, as they have inter-element spacing smaller than half-wavelength, which helps avoid angular ambiguities. However, the uniform degrees of freedom (uDOFs) and array aperture of ULAs are limited, impacting the ability to resolve the number of sources and the accuracy of DOA estimation significantly. Therefore, in order to ensure estimation performance, large-sized ULAs are often employed. Unfortunately, this method not only increases system complexity and hardware costs but also results in significant array mutual coupling effects.

Alternatively, the emergence of sparse arrays [10,11,12,13,14,15,16,17,18,19,20,21,22,23,24,25] provides a direction for solving the problem of ULAs. Via second-order differencing transformation, sparse arrays can obtain virtual ULAs with functionality similar to physical ULAs [10]. Specifically, a sparse array with *M* physical sensors can provide $M^{2}$ virtual sensors, theoretically allowing the identification of sources up to $M^{2}-1$ in number. Additionally, the large inter-element spacing in sparse arrays significantly mitigates array mutual coupling effects.

Typical sparse arrays include the minimum redundant array (MRA) [11], the nested array (NA) [12], and the coprime array (CPA) [13]. MRA features an optimal array geometry, as it offers the maximum contiguous lags for a given number of sensors. However, it lacks closed-form expressions for sensor positions and uDOFs, which necessitates exhaustive searches over all possible spatial combinations of available sensors. Motivated by this, NA and CPA were subsequently proposed. Both NA and CPA can be seen as combinations of two ULAs expanded in different ways. Specifically, one ULA remains fixed, while the inter-element spacing of another ULA is expanded to the number of elements in the first ULA plus one, resulting in the formation of NA. CPA is composed of two sparse ULAs, one consisting of *M* sensors with an inter-element spacing of *N* units, and the other consisting of *N* elements with an inter-element spacing of *M* units, where *M* and *N* are coprime. The sparser nature of CPA results in smaller mutual coupling effects; however, it exhibits a significant number of holes in its difference coarray (DCA), which prevents the full utilization of its virtual elements. In contrast, NA has a completely contiguous lags in its DCA, but the significant mutual coupling effect limits its application to a certain extent. Subsequent research predominantly concentrated on how to maintain the advantages of these two arrays while addressing their corresponding shortcomings. For instance, coprime array with displaced subarrays (CADiS) [14], enhanced CACIS [15], thinned coprime array (TCPA) [16], padded coprime array (PCPA) [17], and *k* times extended coprime array (kECPA) [18] have partially filled the holes in the DCA of CPA through various methods, thereby increasing its uDOFs. However, these strategies still do not achieve a hole-free DCA. Additionally, the generalized nested array (GNA) [19], enhanced nested array (ENA) [20], improved nested array (INA) [21], Iizuka NA [22], super nested array (SuperNA) [23,24], augmented nested array (ANA) [25], and nested array with three sub-ULAs (NA-TS) [26] reconfigured the array geometry of NA to reduce mutual coupling effects and enhance their uDOFs. However, ENA simply divides a dense ULA into two smaller dense ULAs, so its mutual coupling effect is hardly reduced. Meanwhile, INA and the Iizuka NA retain the dense subarray of the prototype NA and suffer from severe mutual coupling effects. Although SuperNA and GNA significantly reduce mutual coupling, they do not increase its uDOFs. In addition, the DCAs of GNA and ANA both have holes. NA-TS shows some improvement in both metrics, but the improvement is relatively modest.

In this paper, a generalized augmented multi-subarray nested array (GAMSNA-I) and its variant, GAMSNA-II are proposed. GAMSNA-I is obtained by decomposing and reconfiguring the dense ULA of NA into multiple parts, resulting in an increase in uDOFs and a reduction in mutual coupling effects. Considering the existence of a dense ULA within GAMSNA-I, further decomposition and reconfiguration of this part results in GAMSNA-II, which maintains the same uDOFs while significantly reducing mutual coupling effects. Additionally, closed-form expressions for the array configurations and uDOFs of both GAMSNA-I and GAMSNA-II are derived and analyzed. Simulation results are provided to demonstrate their superiority in DOA estimation performance.

The rest of this paper is organized as follows: Section 2 introduces the signal model for sparse arrays, relevant concepts of the DCA, and the DOA estimation process. The process of array configuration for the proposed GAMSNA-I and GAMSNA-II is presented in Section 3. The superior performance of these arrays is demonstrated through simulation results in Section 4. Section 5 provides the conclusion of the paper.

## 2. Preliminaries

### 2.1. Signal Model

Consider a sparse array with *M* sensors, where the set of sensor positions is $S=\{l_{i}d|l_{i}\in L,i=1,2,\cdots ,M\}$. Here, *d* is the unit spacing, usually expressed as λ/2, where λ is the wavelength of the signal. L is a set of integers arranged in ascending order, with the first element serving as a reference point and assigned a value of 0, i.e., $l_{1}=0$. Assume that *K* is far-field uncorrelated narrowband source signals imping on the array, with the DOAs of $\Theta =\{\theta_{k}|k=1,2,\cdots ,K\}$, where $\theta_{k}\in \left(-\pi /2,\pi /2\right)$. Then, at time *t*, the received signal vector of the array is modeled as

(1) $$x\left(t\right)=\mathrm{As}\left(t\right)+n\left(t\right)=\sum_{k}^{K}a(\theta_{k})s_{k}\left(t\right)+n\left(t\right)$$

where $A=[a\left(\theta_{1}\right),a\left(\theta_{2}\right),\cdots ,a\left(\theta_{K}\right)]$ represents the array manifold matrix with the column $a\left(\theta_{k}\right)={\left[1,e^{-j2\pi l_{2}d\sin\theta_{k}/\lambda },\cdots ,e^{-j2\pi l_{M}d\sin\theta_{k}/\lambda }\right]}^{T}$. $s\left(t\right)={\left[s_{1}\left(t\right),s_{2}\left(t\right),\cdots ,s_{K}\left(t\right)\right]}^{T}$ is the source vector, and the power of the k-th source is $p_{k}^{2}$. n(t) denotes zero-mean complex additive Gaussian white noise with variance of $\sigma^{2}I_{M}$, where $I_{M}$ represents the M×M-dimensional identity matrix.

### 2.2. Difference Coarray

The essence of DCA lies in the utilization of second-order statistics, namely the covariance matrix of the received signal [27]. According to (1), the covariance matrix of x(t) is calculated as

(2) $$R_{\mathrm{xx}}=E\left[xx^{H}\right]=AR_{\mathrm{ss}}A^{H}+\sigma^{2}I_{M}\approx \frac{1}{N}\sum_{n=1}^{N}xx^{H}$$

where $R_{\mathrm{ss}}=diag\left(\left[p_{1}^{2},p_{2}^{2},\cdots ,p_{K}^{2}\right]\right)$ is the signal covariance matrix, and *N* represents the number of snapshots.

By vectorizing (2), it can be obtained that

(3) $$Z=vec\left(R_{\mathrm{xx}}\right)=BG+\sigma^{2}W_{M}$$

where $B=A^{*}⊙A$ can be seen as an array manifold matrix, $G=diag\left(R_{\mathrm{ss}}\right)=[p_{1}^{2},p_{2}^{2},\cdots$, $p_{K}^{2}{]}^{T}$ and ⊙ is the Khatri-Rao product. Meanwhile, $W_{M}=vec\left(I_{M}\right)$.

Comparing (1) and (3), it can be revealed that (3) is a received signal vector of the array under a single snapshot condition. In this case, B denotes the array manifold matrix, which is composed of the steering vectors corresponding to the DCA of L. The DCA of L is given by

(4) $$D=\left\{\left(l_{i}-l_{j}\right)|i,j=1,2,\cdots ,M\right\}$$

**Definition 1.**  *(Degrees of freedom, DOFs): Given a sparse array L, the DOFs is the cardinality of its DCA D.*

**Definition 2.**  *(Uniform degrees of freedom, uDOFs): The central continuous part of D is named as U, the uDOFs is defined as the cardinality of U.*

**Definition 3.**  *(Virtual element weight function): The number of repetitions of the element with a value of u in D is represented as the weight function w(u) for that element.*

Therefore, w(u) can be written as

(5) $$w\left(u\right)=\left\{l_{i},l_{j}\in L|l_{i}-l_{j}=u,u\in D\right\}$$

### 2.3. DOA Estimation

By analyzing (3), it can be seen that Z not only includes all the received data corresponding to the virtual ULA but also contains redundant elements. Furthermore, according to Definition 2, there exists a possibility that U is not equal to D; in other words, the DCA may not be fully continuous lags. Therefore, to obtain the covariance data corresponding to the virtual ULA, based on Definitions 2 and 3, Z is subjected to filtering, sorting, and redundancy removal. The processing result of Z is denoted as $\bar{Z}$, and it is evident that its dimension is Q×1, where *Q* stands for the uDOFs.

To construct the covariance matrix of the received signals corresponding to the virtual ULA, the spatial smoothing technique [28] is employed to perform the Toeplitz transformation on $\bar{Z}$, and the result is as follows:

(6) $$R_{v}=\left[\begin{array}{cccc} {\bar{Z}}_{\frac{Q+1}{2}} & {\bar{Z}}_{\frac{Q+1}{2}+1} & \cdots & {\bar{Z}}_{Q}\\ {\bar{Z}}_{\frac{Q+1}{2}-1} & {\bar{Z}}_{\frac{Q+1}{2}} & \cdots & {\bar{Z}}_{Q-1}\\ \vdots & \vdots & \ddots & \vdots \\ {\bar{Z}}_{1} & {\bar{Z}}_{2} & \cdots & {\bar{Z}}_{\frac{Q+1}{2}} \end{array}\right]$$

Subsequently, some covariance matrix-based algorithms can be directly applied to $R_{v}$ for DOA estimation, such as the MUSIC algorithm.

### 2.4. Mutual Coupling

The scenario corresponding to (1) does not consider the mutual coupling effects between physical sensors. However, in practice, the mutual coupling effects between sensors are one of the important factors affecting estimation results and cannot be directly ignored. In the context of the presence of mutual coupling effects, (1) is rewritten as

(7) x(t)=CAs(t)+n(t)

where C is the mutual coupling matrix with a dimension of M×M. According to [29], in the case of a linear array, the expression for C is simplified without the need to consider the influence of various factors such as humidity, operating frequency, adjacent objects, etc. Therefore, C can be approximated as a *B*-banded symmetric matrix as

(8) $${\langle C\rangle }_{l_{i},l_{j}}=\left\{\begin{array}{c} c_{\left|l_{i}-l_{j}\right|},\;if\left|l_{i}-l_{j}\right|\leq B\\ 0,\;elsewhere \end{array}\right.$$

where $l_{i},l_{j}\in L$, and $c_{i},i=0,1,\cdots ,B$ are the coupling coefficients, satisfying $c_{0}=1>|c_{1}\left|>\right|c_{2}\left|>\cdots >\right|c_{B}|$ and $\left|c_{i}/c_{j}\right|=j/i,i,j=0,1,\cdots ,B$.

To objectively evaluate the mutual coupling effects, the coupling leakage is introduced, which is represented as

(9) $$L=\frac{\left||C-diag\left(C\right)\right|{|}_{F}}{\left||C\right|{|}_{F}}$$

where $\left|\right|\cdot {\left|\right|}_{F}$ is the Frobenius norm of a matrix. *L* reflects the energy proportion of the off-diagonal components of C, and a smaller value indicates weaker mutual coupling effects between elements in the sensor array.

## 3. Proposed Array Configurations

In this section, the structural compositions of the proposed GAMSNA-I and GAMSNA-II are primarily introduced, along with their closed-form expressions of sensor positions and achievable uDOFs. By reconfiguring two subarray elements of the prototype NA, GAMSNA-I is proposed, increasing uDOFs while simultaneously reducing mutual coupling influences. Furthermore, to further mitigate the impact of mutual coupling effects while maintaining uDOFs, improvements are made to GAMSNA-I to obtain GAMSNA-II.

### 3.1. GAMSNA-I

The normalized set of element positions of the prototype NA, formed by two subarrays belonging to a dense ULA and a sparse ULA respectively, is denoted as

(10) $$L_{NA}=\left\{m_{1},0\leq m_{1}\leq M_{1}-1\right\}\cup \left\{m_{2}\left(M_{1}+1\right)+M_{1},0\leq m_{2}\leq M_{2}-1\right\}$$

where $M_{1}$ and $M_{2}$ represent the number of sensors in two subarrays, respectively, which satisfies the optimal configuration of NA, as shown in Table 1.

The mutual coupling between the elements of NA primarily arises from the dense ULA portion. Therefore, the main idea of GAMSNA-I is to reconfigure the structure of the dense ULA and make appropriate adjustments to the sparse ULA, achieving an increase in uDOFs while reducing mutual coupling.

The structure of GAMSNA-I consists of four parts, represented as

(11) $$L_{GAMSNA-I}=L_{1}\cup L_{2}\cup L_{3}\cup L_{4}$$

where the closed-form expressions of $L_{1}$, $L_{2}$, $L_{3}$, and $L_{4}$ are

(12) $$\left\{\begin{array}{c} L_{1}=\left\{0,1,M_{1}\right\},M_{1}\geq 4\\ L_{2}=\left\{3+\ell \left(M_{1}+1\right)|\ell =0,1,\cdots ,M_{2}-1\right\}\\ L_{3}=\left\{M_{2}\left(M_{1}+1\right)-M_{1}+3\right\}\\ L_{4}=\left\{\left(M_{1}+1\right)\left(M_{2}+1\right)-M_{1}+2-\eta |\eta =1,2,\cdots ,M_{1}-4\right\} \end{array}\right.$$

Note in (12) that $M_{1}\geq 4$. Combining this with Table 1, it can be deduced that the minimum number of sensors in GAMSNA-I is 8, i.e., M≥8. Additionally, when $M_{1}=4$, i.e., M=8 or M=9, $L_{4}=0$, the array aperture of GAMSNA-I is equal to that of NA, and both arrays have a DCA without holes, resulting in equal uDOFs for both. This implies that the advantage of GAMSNA-I in this case is the relatively less mutual coupling. When $M_{1}=5$, i.e., M=10 or M=11, $L_{4}=1$, although the array aperture of GAMSNA-I is larger than that of NA, there are holes in the DCA of GAMSNA-I, and the uDOFs of GAMSNA-I are less than those of NA. In this case, GAMSNA-I sacrifices two virtual array elements to reduce mutual coupling.

When M≥12, GAMSNA-I demonstrates advantages both in terms of uDOFs and mutual coupling. The array aperture of GAMSNA-I is larger than that of NA, and its DCA is hole-free, as summarized in Proposition 1.

**Proposition 1.**  *The DCA of the GAMSNA-I as defined in (12) is a hole-free ULA, i.e., $D=U=[-Q_{g},Q_{g}]$ with $Q_{g}=\left(\left\lfloor \frac{M}{2}\right\rfloor +1\right)\left\lceil \frac{M}{2}\right\rceil +2$.*

**Proof of Proposition 1.**  Refer to Appendix A. □

From Proposition 1, Corollary 1 can be obtained.

**Corollary 1.**  *For the GAMSNA-I with M sensors, the closed-form expression of uDOFs is $uDOFs=\frac{M^{2}}{2}+M+5$ in the case of M being even, and $uDOFs=\frac{M^{2}-1}{2}+M+6$ in the case of M being odd.*

**Proof of Corollary 1.**  According to (12) and Proposition 1, it follows that

(13) $$uDOFs=2Q_{g}+1=2\left(\left\lfloor \frac{M}{2}\right\rfloor +1\right)\left\lceil \frac{M}{2}\right\rceil +5$$

In the case of *M* being even, $\left\lfloor \frac{M}{2}\right\rfloor =\left\lceil \frac{M}{2}\right\rceil =\frac{M}{2}$, so $uDOFs=\frac{M^{2}}{2}+M+5$. In the case of *M* being odd, $\left\lfloor \frac{M}{2}\right\rfloor =\frac{M-1}{2},\left\lceil \frac{M}{2}\right\rceil =\frac{M+1}{2}$, so $uDOFs=\frac{M^{2}-1}{2}+M+6$. Therefore, the proof is completed. □

**Figure 1:**
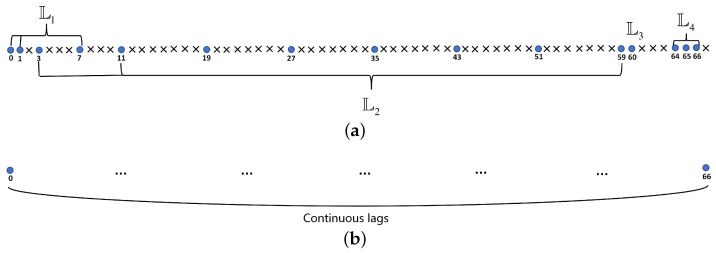
An example of GAMSNA-I with M=15, and the positive result of its DCA. (**a**) Sensor positions of GAMSNA-I. (**b**) Positive DCA of GAMSNA-I.

Figure 1a illustrates the structural composition of GAMSNA-I with M=15, specifically $L_{1}=\left\{0,1,7\right\}$, $L_{2}=\left\{3,11,19,27,35,43,51,59\right\}$, $L_{3}=\left\{60\right\}$, $L_{4}=\left\{64,65,66\right\}$. Figure 1b shows the positive result of DCA for GAMSNA-I, which is hole-free.

Corollary 1 indicates that the improved uDOFs are greater than those of NA. Moreover, according to [30], it is known that w(1), w(2) and w(3) dominate the mutual coupling. Based on the analysis of (12), for M≥12, the first three weight functions of GAMSNA-I are

(14) $$\left\{\begin{array}{c} w\left(1\right)=M_{1}-3\\ w\left(2\right)=M_{1}-5\\ w\left(3\right)=\left\{\begin{array}{cc} 2, & M_{1}=6\\ M_{1}-6, & M_{1}>6 \end{array}\right. \end{array}\right.$$

Compared to the first three weight functions of NA, which are $w\left(1\right)=M_{1}$, $w\left(2\right)=M_{1}-1$ and $w\left(3\right)=M_{1}-2$, the results of (14) demonstrate that the mutual coupling of GAMSNA-I is less. Nevertheless, there is a linear increase in the correlation between the weight functions of GAMSNA-I and the number of sensors. As the number of sensors increases, the impact of mutual coupling becomes more significant, imposing substantial limitations on the accuracy improvement of DOA estimation. Therefore, there is a need to improve GAMSNA-I to reduce its mutual coupling.

### 3.2. GAMSNA-II

According to (12), it can be noted that $L_{4}$ is a dense ULA with strong mutual coupling. Therefore, on the basis of GAMSNA-I, by adjusting some sensor positions of $L_{4}$ to reduce mutual coupling, GAMSNA-II is obtained.

The composition of GAMSNA-II is as follows:

(15) $$\left\{\begin{array}{c} {L^{\prime }}_{1}=\left\{0,1,M_{1}\right\},M_{1}\geq 4\\ {L^{\prime }}_{2}=\left\{3+\ell^{\prime }\left(M_{1}+1\right)|\ell^{\prime }=0,1,\cdots ,M_{2}-1\right\}\\ {L^{\prime }}_{3}=\left\{M_{2}\left(M_{1}+1\right)-M_{1}+3\right\}\\ {L^{\prime }}_{4}=\left\{\left(M_{1}+1\right)\left(M_{2}+1\right)-M_{1}+2-\eta^{\prime }|\eta^{\prime }\in \left[1,M_{1}-4\right],\eta^{\prime }\;\mathrm{is}\;\mathrm{odd}.\right\}\\ {L^{\prime }}_{5}=\left\{\left(M_{1}+1\right)\left(M_{2}+1\right)+3-v_{5}^{\prime }|v_{5}^{\prime }\in \left[1,M_{1}-4\right],v_{5}^{\prime }\;\mathrm{is}\;\mathrm{even}.\right\} \end{array}\right.$$

**Figure 2:**
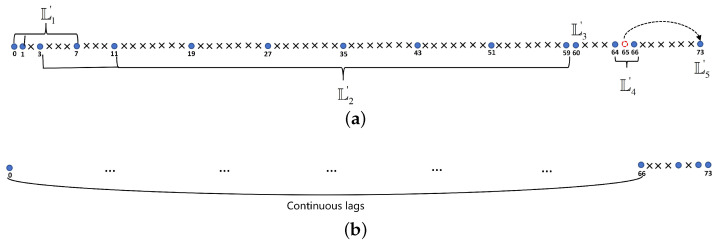
An example of GAMSNA-II with M=15, and the positive result of its DCA. (**a**) Sensor positions of GAMSNA-II. (**b**) Positive DCA of GAMSNA-II.

Note that GAMSNA-II is only feasible with $|L_{4}|\geq 3$, i.e., M≥14. Figure 2 shows the array structure of GAMSNA-II. It is worth noting that despite the presence of holes in the DCA of GAMSNA-II, the uDOFs corresponding to the central ULA portion of the DCA remain consistent with those of GAMSNA-I.

**Proposition 2.**  *The continuous part of the DCA for GAMSNA-II defined in (15) is equal to that of GAMSNA-I, i.e., $U_{GAMSNA-II}=U_{GAMSNA-I}=\left[-Q_{g},Q_{g}\right]$ with $Q_{g}=\left(\left\lfloor \frac{M}{2}\right\rfloor +1\right)\left\lceil \frac{M}{2}\right\rceil +2$.*

**Proof of Proposition 2.**  Refer to Appendix B. □

It can be inferred from (15) that the first three weight functions of GAMSNA-II are

(16) $$\left\{\begin{array}{c} w(1)=2\\ w\left(2\right)=M_{1}-5\\ w\left(3\right)=\left\{\begin{array}{cc} 2, & M_{1}=6\\ 1, & M_{1}>6 \end{array}\right. \end{array}\right.$$

Compared with GAMSNA-I, the mutual coupling of GAMSNA-II is less, and as the number of sensors increases, their differences become increasingly apparent. In addition, the expressions of uDOFs and sensor number for different array configurations are compared in Table 2, where $M_{i}$ represents the number of sensors in subarray $L_{i}$ of the corresponding sensor array.

## 4. Numerical Simulations

In this section, numerical simulations are performed to assess the performance of the proposed GAMSNA-I and GAMSNA-II from three key aspects: uDOFs, coupling leakage, and DOA estimation accuracy.

### 4.1. uDOFs

**Figure 3:**
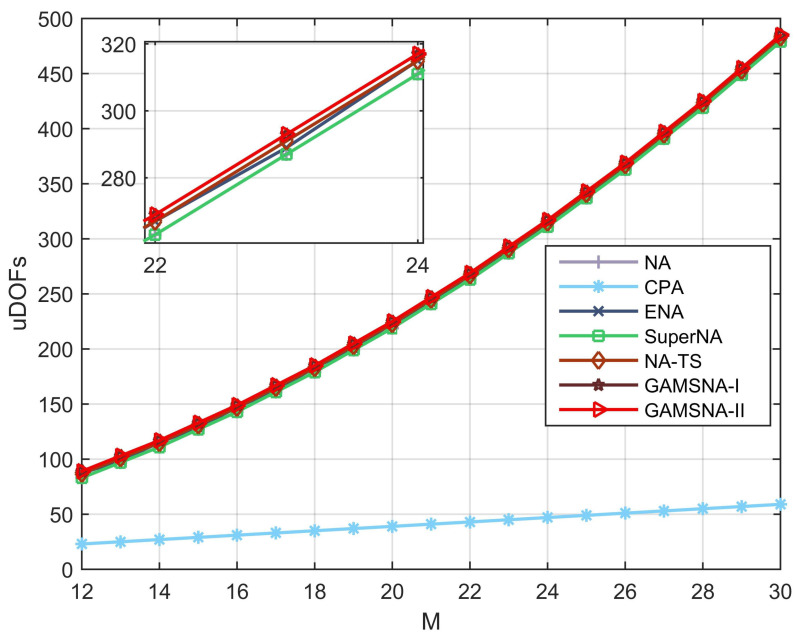
uDOFs versus *M* for different arrays.

In the first numerical simulation, we compare the uDOFs of CPA, NA, SuperNA, ENA, NA-TS, GAMSNA-I and GAMSNA-II. The numerical relationship between the uDOFs of different arrays and the sensor quantity *M* is shown in Figure 3, where *M* = [14:1:30]. It can be observed that due to the presence of a large number of holes, the uDOFs curve for CPA is the lowest and considerably lower than the other curves. The uDOFs curves of SuperNA and NA overlap, and they are lower compared to the uDOFs curves of other NA-type arrays, indicating that SuperNA does not have an advantage over NA in terms of uDOFs and performs worse than other NA-type arrays. The structural uniqueness of ENA makes its uDOFs dependent on the parity of *M*. Specifically, when *M* is even, its uDOFs is greater than that of NA, while when *M* is odd, its uDOFs is equal to that of NA. The uDOFs curve for NA-TS is higher than the uDOF curves for the first three NA-type arrays, indicating its superior uDOFs performance. Finally, the uDOFs curves for the proposed GAMSNA-I and GAMSNA-II overlap with each other and are higher than the other curves, suggesting that these two array configurations exhibit the same excellent performance in terms of uDOFs. These results validate the effectiveness of the uDOFs property summarized for different array configurations in Table 2.

### 4.2. Coupling Leakage

In the second numerical simulation, we compare the weight functions and coupling leakage of CPA, NA, SuperNA, ENA, NA-TS, MISC, GENAMS, GAMSNA-I, and GAMSNA-II. In the interest of fairness, consider that these arrays uniformly consist of 15 sensors, with the specific sensor positions listed in Table 3.

**Figure 4:**
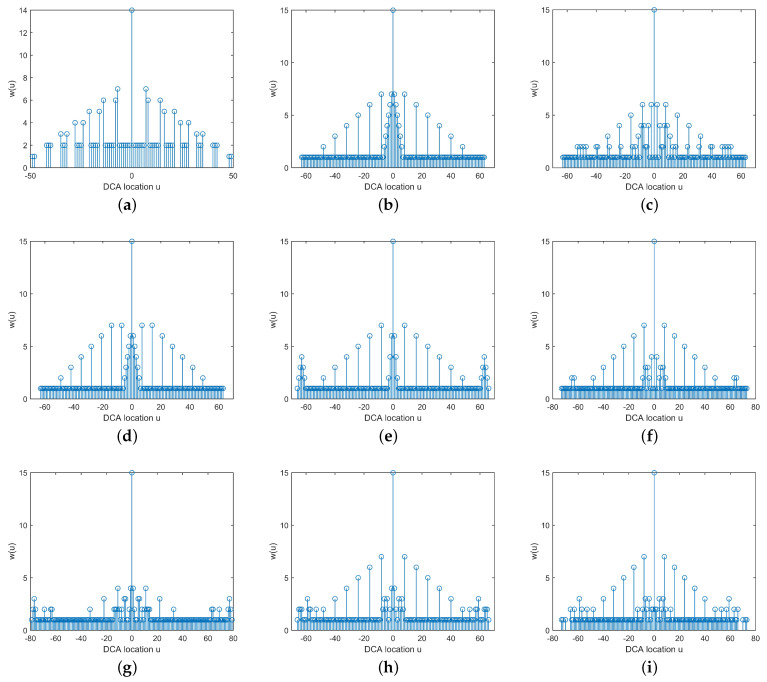
The virtual element weight functions of nine kinds of 15-element sparse arrays. (**a**) CPA. (**b**) NA. (**c**) Super NA. (**d**) ENA. (**e**) NA-TS. (**f**) MISC. (**g**) GENAMS. (**h**) GAMSNA-I. (**i**) GAMSNA-II.

According to Definition 3, the results of the virtual element weight functions for seven kinds of arrays are shown in Figure 4. To minimize the impact of mutual coupling effects as much as possible, weight functions with larger numerical values should be located far away from u=0. By observing Figure 4a, it can be noted that the weight function values near u=0 are all 1, indicating that the mutual coupling effect of CPA is relatively small. In Figure 4b, a large number of weight functions with large numerical values are concentrated near u=0, reflecting the significant mutual coupling in NA. Subsequent Figure 4c–i disperse weight functions with large numerical values to varying degrees, away from u=0, thus confirming that their corresponding arrays exhibit reduced mutual coupling compared to NA. By comparison, it can be observed that MISC and the proposed GAMSNA-II have fewer weight functions with large numerical values near u=0, and therefore, it can be concluded that they have the least mutual coupling among all array configurations being compared. To provide a more intuitive representation, Table 4 presents a numerical comparison of the first three weight functions, and coupling leakage. Note that the values of the coupling coefficients are $c_{1}=0.3e^{\left(j\pi /3\right)}$, $c_{i}=c_{1}e^{\left[-j(i-1)\pi /8\right]}/i,2\leq i\leq B$, where B=100 [31].

### 4.3. DOA Estimation Performance

The DOA estimation performances of various array configurations are compared in this subsection using the MUSIC algorithm, where the peak search step size is set to 0.1°. The array configurations are the same as those given in Table 3.

#### 4.3.1. MUSIC Spectrum

This experiment involves comparing the MUSIC spectrum of CPA, NA, SuperNA, ENA, NA-TS, MISC, GENAMS, GAMSNA-I, and GAMSNA-II under the scenario where there are 13 sources with DOAs of $\Theta ={\left[-30:5:30\right]}^{\circ }$, a signal-to-noise ratio (SNR) of 0 dB, and a snapshot number of 500.

**Figure 5:**
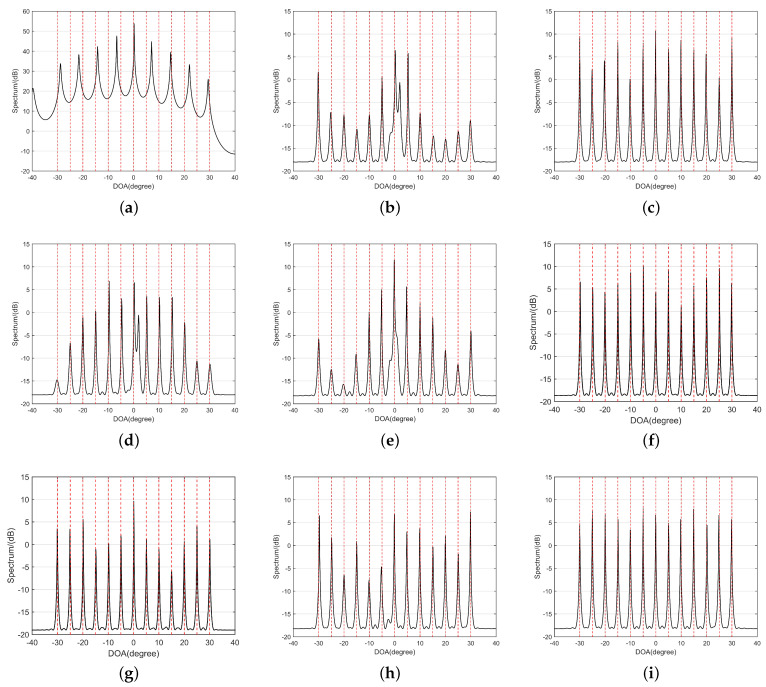
MUSIC spectrum of nine kinds of 15-element sparse arrays. (**a**) CPA. (**b**) NA. (**c**) Super NA. (**d**) ENA. (**e**) NA-TS. (f) MISC. (**g**) GENAMS. (**h**) GAMSNA-I. (**i**) GAMSNA-II.

The results are presented in Figure 5, where the red dashed lines and the black solid lines, respectively, indicate the true DOAs and the estimated spectra. In Figure 5a, the number of peaks in the spectral curve is inconsistent with the number of sources, and the actual values and estimated values hardly match, indicating poor performance of CPA in this experiment. Furthermore, when observing Figure 5b,d, it can be noticed that although the estimated DOA values are near the true values, there are perturbations in the peaks. This is due to significant mutual coupling of NA and ENA, which will lead to significant errors in the final DOA estimation results. In Figure 5e, although there are no peak disturbances, the peaks are not sharp enough. This is because the larger uDOFs of NA-TS balance the effects of mutual coupling, but the degree of balance is relatively low. Based on the observations from Figure 5c,f–i, it is evident that the DOA estimation results for SuperNA, MISC, GENAMS, GAMSNA-I, and GAMSNA-II are notably accurate. This is consistent with their own high uDOFs and low mutual coupling, as detailed in Table 4. Among these, due to the fact that the mutual coupling of GAMSNA-I is higher than SuperNA, although it has greater uDOFs, the sharpness of its spectral curve peaks is still inferior to SuperNA. The significantly high uDOFs of GENAMS balance the influence of mutual coupling well, making its MUSIC spectrum sharper than GAMSNA-I, but still inferior to SuperNA. This result suggests that, when the difference in uDOFs is within a certain range, lower mutual coupling can appropriately compensate for the disadvantages of uDOFs. The DOA estimation results of MISC are superior to that of GENAMS, which also confirms this conclusion. Additionally, when comparing Figure 5f,i, it can be observed that the peak stability of the spectral curves for MISC and GAMSNA-II is essentially consistent. This suggests that both exhibit similar low mutual coupling effects, ultimately resulting in similar DOA estimation outcomes.

#### 4.3.2. Root Mean Square Error Performance

The next simulation compares the root mean square error (RMSE) performance of CPA, NA, SuperNA, ENA, NA-TS, GAMSNA-I, and GAMSNA-II versus the input SNR and the number of snapshots. The fixed parameter settings are N=500 snapshots, SNR=0 dB, and K=13 sources with $\theta_{k}∼U\left(-90^{\circ },90^{\circ }\right)$, where U(·) means uniform distribution. RMSE is expressed as follows:

(17) $$R\;M\;S\;E=\sqrt{\frac{1}{CK}(\sum_{c=1}^{C}\sum_{k=1}^{K}{\left(\theta_{k}-{\hat{\theta }}_{k,c}\right)}^{2}},$$

where *C* represents the number of Monte Carlo trials, and it is set to 500 in this subsection.

**Figure 6:**
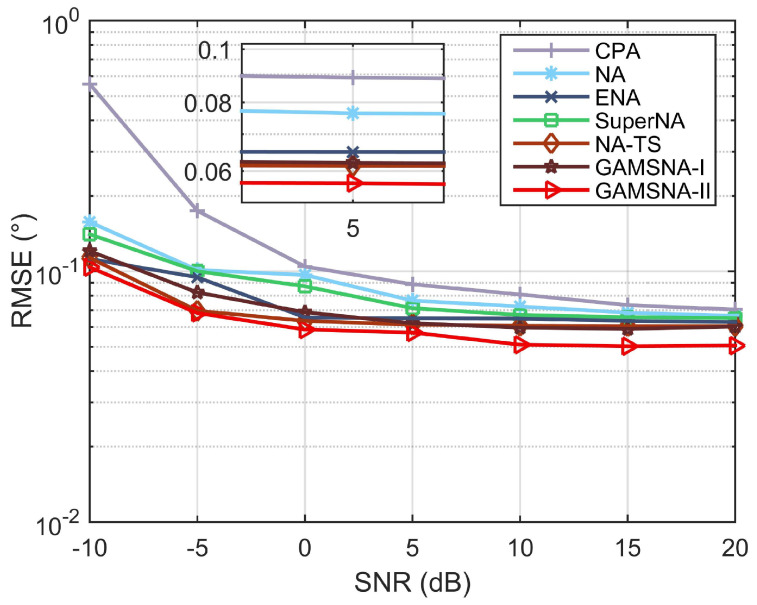
RMSE performance versus input SNR.

RMSE performance versus the input SNR is shown in Figure 6, where SNR=[−10:5:20] dB. We observe that throughout the entire SNR range, the proposed GAMSNA-I and GAMSNA-II exhibit better performance than other arrays. Among these, due to the smaller mutual coupling effects, the advantage of GAMSNA-II is more pronounced, as evidenced by its lower RMSE curve. The results indicate that the performance of these two arrays is superior to other arrays, with GAMSNA-II demonstrating the best performance.

**Figure 7:**
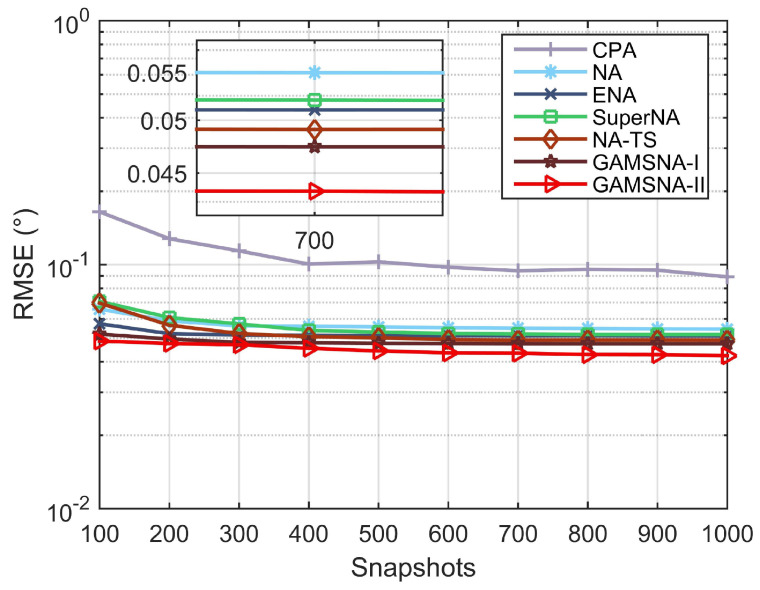
RMSE performance versus number of snapshots.

Figure 7 illustrates RMSE performance versus the number of snapshots, with the set of snapshot numbers as [100:100:1000]. By observation, it can be noticed that when the number of snapshots is less than 300, the differences in RMSE performance among the arrays are relatively pronounced, and the RMSE curves of GAMSNA-I and GAMSNA-II are lower than the others. As the number of snapshots increases—except for GAMSNA-II, which exhibits a relatively significant improvement in RMSE performance—the RMSE performance of the other arrays remains relatively constant with minimal differences between them. Throughout the experiment, GAMSNA-II consistently outperforms GAMSNA-I in RMSE performance. The above-mentioned phenomenon indicates that the proposed GAMSNA-I and GAMSNA-II exhibit superior DOA estimation performance, with GAMSNA-II demonstrating lower mutual coupling, thus offering a significant advantage in accurately estimating DOA.

## 5. Conclusions

In this paper, a generalized augmented multi-subarray nested array (GAMSNA-I) with a large number of uDOFs and small mutual coupling is proposed. Additionally, its variant, GAMSNA-II, is developed to further reduce mutual coupling. The array configuration and uDOFs of GAMSNA-I are described by closed-form expressions constrained by the number of sensors. Considering the existence of a dense ULA subarray in GAMSNA-I, GAMSNA-II is developed with a sparser array configuration, while its uDOFs is proven to remain unaltered. Theoretical analysis and numerical simulation results demonstrate the effectiveness and superiority of the proposed GAMSNA-I and GAMSNA-II in terms of uDOFs, coupling leakage, spectral estimation, and DOA estimation accuracy.

## Figures and Tables

**Table 1:** Optimal sensor quantity configuration for two subarrays of NA.

The Total Number of Sensors	Subarray 1	Subarray 2
*M*	$M_{1}$	$M_{2}$
Odd	(M−1)/2	(M+1)/2
Even	M/2	M/2

**Table 2:** The relationship between the number of sensors and the uDOFs for seven types of sparse arrays.

Array Configuration	Sensor Number	uDOFs
CPA	M+N	2(M+N)−1
NA	$M_{1}+M_{2}$	$2M_{2}\left(M_{1}+1\right)-1$
SuperNA	$M_{1}+M_{2}$	$2M_{2}\left(M_{1}+1\right)-1$
ENA	$M_{1}+M_{2}$	$2M_{1}\left(M_{2}+1\right)+1$
NA-TS	even: $\begin{array}{c} M_{1}=\left(M-2M_{3}\right)/2,\;M_{2}=M/2,\\ 1\leq M_{3}<M/2 \end{array}$	$M^{2}/2+2M-2M_{3}-1$
	odd: $\begin{array}{c} M_{1}=\left(M-2M_{3}-1\right)/2,\;M_{2}=\\ \left(M+1\right)/2,\;1\leq M_{3}<\left(M-1\right)/2 \end{array}$	$M^{2}/2+2M-2M_{3}-3/2$
GAMSNA-I	even: $\begin{array}{c} M_{1}=3,\;M_{2}=M/2,\;M_{3}=1,\\ M_{4}=M/2-4,\;M\geq 12 \end{array}$	$M^{2}/2+M+5$
	odd: $\begin{array}{c} M_{1}=3,\;M_{2}=\left(M+1\right)/2,\;M_{3}=1,\\ M_{4}=\left(M+1\right)/2-5,\;M\geq 12 \end{array}$	$(M^{2}-1)/2+M+6$
GAMSNA-II	even: $\begin{array}{c} M_{1}=3,\;M_{2}=M/2,\;M_{3}=1,\\ M_{4}=\left\lceil M/4-2\right\rceil ,\;M_{5}=M/2-\\ 4-M_{4},\;M\geq 14 \end{array}$	$M^{2}/2+M+5$
	odd: $\begin{array}{c} M_{1}=3,\;M_{2}=\left(M+1\right)/2,\;M_{3}=1,\\ M_{4}=\left\lceil (M+1)/4-5/2\right\rceil ,\;M_{5}=\\ \left(M+1\right)/2-5-M_{4},\;M\geq 14 \end{array}$	$(M^{2}-1)/2+M+6$

**Table 3:** Sensors locations for nine sparse arrays.

Array Configuration	Sensor Locations
CPA	{0, 7, 8, 14, 16, 21, 24, 28, 32, 35, 40, 42, 48, 49}
NA	{0, 1, 2, 3, 4, 5, 6, 7, 15, 23, 31, 39, 47, 55, 63}
SuperNA	{0, 2, 4, 6, 9, 11, 13, 15, 23, 31, 39, 47, 55, 62, 63}
ENA	{0, 1, 2, 3, 4, 5, 6, 14, 21, 28, 35, 42, 49, 56, 63}
NA-TS	{0, 1, 2, 3, 11, 19, 27, 35, 43, 51, 59, 63, 64, 65, 66}
MISC	{0, 1, 6, 14, 22, 30, 38, 46, 54, 62, 64, 66, 69, 71, 73}
GENAMS	{0, 1, 2, 5, 10, 15, 26, 37, 48, 59, 65, 71, 77, 78, 79}
GAMSNA-I	{0, 1, 3, 7, 11, 19, 27, 35, 43, 51, 59, 60, 64, 65, 66}
GAMSNA-II	{0, 1, 3, 7, 11, 19, 27, 35, 43, 51, 59, 60, 64, 66, 73}

**Table 4:** A summary of the uDOFs, virtual element weight functions, and coupling leakage for nine types of sparse arrays with 15 sensors.

**Array Configuration**	CPA	NA	SuperNA	ENA	NA-TS
uDOFs	29	127	127	127	133
*w*(1)	2	7	1	6	6
*w*(2)	2	6	6	5	4
*w*(3)	2	5	1	4	2
L	0.2054	0.3236	0.1970	0.3010	0.2894
**Array Configuration**	MISC	GENAMS	GAMSNA-I	GAMSNA-II	
uDOFs	147	159	133	133	
*w*(1)	1	4	4	2	
*w*(2)	4	2	2	2	
*w*(3)	1	1	1	1	
L	0.1772	0.2368	0.2434	0.1928	

## Data Availability

Data are contained within the article.

## Appendix A. Proof of Proposition 1

Since D is symmetric about position zero, for simplifying the proof steps, only the positive part of D is proven to be continuous. Defining symbol $D_{i,j}=L_{i}-L_{j},i\geq j$, according to (12), the DCAs between the four subarrays are

(A1) $$\left\{\begin{array}{c} D_{11}=\left\{0,1,M_{1}-1,M_{1}\right\}\\ D_{21}=\left\{3+\ell \left(M_{1}+1\right),2+\ell \left(M_{1}+1\right),\left(3-M_{1}\right)+\ell \left(M_{1}+1\right)|\ell =\left[0,M_{2}-1\right]\right\}\\ D_{31}=\left\{\left(M_{2}-1\right)\left(M_{1}+1\right)+4,\left(M_{2}-1\right)\left(M_{1}+1\right)+3,\left(M_{2}-2\right)\left(M_{1}+1\right)+5\right\}\\ D_{41}=\left\{M_{2}\left(M_{1}+1\right)+3-\eta ,M_{2}\left(M_{1}+1\right)+2-\eta ,\left(M_{2}-1\right)\left(M_{1}+1\right)+4-\eta |\eta =\left[1,M_{1}-4\right]\right\}\\ D_{22}=\left\{\ell \left(M_{1}+1\right)|\ell =\left[0,M_{2}-1\right]\right\}\\ D_{32}=\left\{\left(M_{2}-\ell \right)\left(M_{1}+1\right)-M_{1}|\ell =\left[0,M_{2}-1\right]\right\}\\ D_{42}=\left\{\left(M_{2}-\ell \right)\left(M_{1}+1\right)-\eta |\ell =\left[0,M_{2}-1\right],\eta =\left[1,M_{1}-4\right]\right\}\\ D_{33}=\left\{0\right\}\\ D_{43}=\left\{M_{1}-\eta |\eta =\left[1,M_{1}-4\right]\right\}\\ D_{44}=\left\{\left[0,M_{1}-5\right]\right\} \end{array}\right.$$

Since $L_{2}$ is a set of arithmetic sequences with a common difference of $M_{1}+1$, and the last element of $L_{1}$, which is $M_{1}$, falls between the first and second elements of $L_{2}$, it is only necessary to ensure continuity from 0 to $M_{1}+4$ to guarantee continuity from 0 to $3+\left(M_{2}-1\right)\left(M_{1}+1\right)$.

When ℓ=0, the combination of $D_{11}$ and $D_{21}$ contains a continuous range [0,3]. $D_{21}$ has ‘4’ (when ℓ=1). Considering $\ell =M_{2}-1$, $D_{42}$ has a continuous subset $\left[5,M_{1}\right]$. $D_{22}$ contains ‘$M_{1}+1$’. Let $\ell =M_{2}-2$; the element of $D_{32}$ is ‘$M_{1}+2$’. In addition, $D_{21}$ includes ‘$M_{1}+3$’ and ‘$M_{1}+4$’ (when ℓ=1). In summary, the continuous range $\left[0,M_{1}+4\right]$ can be obtained through $D_{11}$, $D_{21}$, $D_{22}$, $D_{32}$ and $D_{42}$, that is, the continuous set $\left[0,3+\left(M_{2}-1\right)\left(M_{1}+1\right)\right]$ can be obtained. Furthermore, $D_{31}$ contains ‘$4+\left(M_{2}-1\right)\left(M_{1}+1\right)$’. In $D_{42}$, let ℓ=0 obtain the continuous part $\left[5+\left(M_{2}-1\right)\left(M_{1}+1\right),M_{2}\left(M_{1}+1\right)-1\right]$. The continuous range of $\left[M_{2}\left(M_{1}+1\right),M_{2}\left(M_{1}+1\right)+2\right]$ can be provided by $D_{41}$. Thus, the continuous range $\left[0,M_{2}\left(M_{1}+1\right)+2\right]$ exists, which completes the proof.

## Appendix B. Proof of Proposition 2

The ${L^{\prime }}_{4}$ and ${L^{\prime }}_{5}$ of GAMSNA-II originate from the reallocation of the $L_{4}$ of GAMSNA-I. Specifically, ${L^{\prime }}_{4}$ is the odd-numbered part of η in $L_{4}$, while ${L^{\prime }}_{5}$ is the even-numbered part of η in $L_{4}$ plus $M_{1}+1$. Therefore, it is only necessary to prove that ${L^{\prime }}_{5}$ has the same effect as the even-numbered part of η in $L_{4}$. For simplicity, only the expressions for ${D^{\prime }}_{42}$, ${D^{\prime }}_{43}$ and ${D^{\prime }}_{52}$ are given.

(A2) $$\left\{\begin{array}{c} {D^{\prime }}_{42}=\left\{\left(M_{2}-\ell^{\prime }\right)\left(M_{1}+1\right)-\eta^{\prime }\right|\ell^{\prime }=\left[0,M_{2}-1\right],\eta^{\prime }=\left[1,M_{1}-4\right],\eta^{\prime }\;\mathrm{is}\;\mathrm{odd}\}\\ {D^{\prime }}_{43}=\left\{M_{1}-\eta^{\prime }\right|\eta^{\prime }=\left[1,M_{1}-4\right],\eta^{\prime }\;\mathrm{is}\;\mathrm{odd}\}\\ {D^{\prime }}_{52}=\left\{\left[M_{2}-\left(\ell^{\prime }-1\right)\right]\left(M_{1}+1\right)-\eta^{\prime }\right|\ell^{\prime }=\left[0,M_{2}-1\right],\eta^{\prime }=\left[1,M_{1}-4\right],\eta^{\prime }\;\mathrm{is}\;\mathrm{even}\} \end{array}\right.$$

It is easy to know that $\ell^{\prime }-1=\left[-1,M_{2}-2\right]$. So, when $\eta^{\prime }$ is even, the elements that $D_{42}$ has but ${D^{\prime }}_{52}$ does not have are $\left(M_{1}+1\right)-\eta^{\prime }$. It is worth noting that ${D^{\prime }}_{43}$ contains all the elements of $\left\{\left(M_{1}+1\right)-\eta^{\prime }|\eta^{\prime }\;is\;even\right\}$. Therefore, when considering the contribution to ensure that continuities of DCA, ${D^{\prime }}_{42}$, ${D^{\prime }}_{52}$, and ${D^{\prime }}_{43}$ are equivalent to $D_{42}$. It is worth noting that ${D^{\prime }}_{43}$ is a subset of $D_{43}$, which was not involved in Proof of Proposition 1. Therefore, the use of elements of ${D^{\prime }}_{43}$ in this proof does not have an impact on the continuity of DCA. In conclusion, the continuous part of the DCA of GAMSNA-II is completely equal to that of GAMSNA-I.
